# Cytogenetic Characterization of the Yak (*Bos grunniens*) Prometaphase Chromosomes and Comparison with Cattle (*Bos taurus*)

**DOI:** 10.3390/genes17080943

**Published:** 2026-08-13

**Authors:** Alfredo Pauciullo, Davide Nicodemo, Neyrouz Letaief, Halina Černohorská, Svatava Kubičková, Miluše Vozdová, Pietro Parma, Leopoldo Iannuzzi, Gianfranco Cosenza

**Affiliations:** 1 Department of Agricultural, Forest and Food Sciences, University of Torino, Largo Paolo Braccini 2, 10095 Grugliasco, Italy; neyrouz.letaief@unito.it; 2 Directorate for Agriculture and Rural Development, European Commission, Rue de La Loi 130, 1000 Brussels, Belgium; 3 Veterinary Research Institute, Hudcova 296/70, 62100 Brno, Czech Republic; halina.cernohorska@vri.cz (H.Č.); svatava.kubickova@vri.cz (S.K.); miluse.vozdova@vri.cz (M.V.); 4 Department of Agriculture and Environmental Sciences, University of Milano, Via Celoria 2, 20133 Milano, Italy; pietro.parma@unimi.it; 5 Institute for the Animal Production System in the Mediterranean Environment, National Research Council of Italy, Piazzale Enrico Fermi 1, 80055 Portici, Italy; leopoldo.iannuzzi@ispaam.cnr.it; 6 Department of Agriculture, University of Napoli Federico II, Piazza Carlo di Borbone 1, 80055 Portici, Italy; giacosen@unina.it

**Keywords:** *Bos grunniens*, *Bos taurus*, karyotype, high-resolution banding, sister chromatid exchange (SCE), nucleolus organizer regions (NORs), zoo-FISH, BAC-FISH

## Abstract

Background/Objectives: The domestic yak (*Bos grunniens*) is a livestock species of major relevance in high-altitude environments and an important model for studying adaptation and reproductive isolation within *Bovini*. Despite its close phylogenetic relationship with cattle (*Bos taurus*), yak × cattle hybrids show a marked sex-biased fertility pattern, with fertile females and generally sterile F1 males, suggesting that subtle chromosomal or genomic differences may underlie post-zygotic reproductive barriers. In this study, we performed a cytogenetic characterization of eight adult yak bulls imported and reared in Central Italy using conventional and molecular cytogenetic approaches. Results: GTG-, RBG-, RBA- and CBA-banding confirmed the yak diploid number as 2n = 60 and the fundamental number as NF = 62, with banding patterns highly comparable to the standardized cattle karyotype. CBA-banding showed an X chromosome lacking evident constitutive heterochromatin and a Y chromosome with distal C-positive blocks. Chromosome instability was low, with 3.75% abnormal metaphases, mainly represented by chromatid and iso-chromatid breaks, while the mean sister chromatid exchange (SCE) rate was 5.19 ± 2.14 per cell. Sequential Ag-NOR/RBA staining localized nucleolar organizer regions (NORs) at the telomeres of autosomes 2, 3, 4, 11 and 25, as in cattle. Zoo-FISH using bovine chromosome paints for X, Y, 5 and 15 showed complete hybridization to the corresponding yak chromosomes, and BAC-FISH mapped the Y-linked *ZFY* and *SRY* genes to positions homologous to those reported in cattle. A comparative bioinformatics analysis of available yak genome assemblies confirmed the overall genome-wide correspondence with cattle, while revealing chromosome orientation issues and small local inconsistencies that may be relevant for comparative mapping and probe design. Conclusions: Overall, at the resolution tested, these findings support broad macrostructural conservation of yak and cattle karyotypes and provide cytogenetic reference data for yak populations reared outside of their traditional range. The persistence of F1 male sterility despite this large-scale chromosomal conservation suggests that fine-scale sex chromosome differences, particularly involving pseudoautosomal regions, recombination boundaries, or heterochromatin organization, may deserve targeted investigation.

## 1. Introduction

The domestic yak (*B. grunniens*) is a key livestock species for human communities living at high altitudes in Central and Inner Asia, providing meat, milk, fiber, draught power and manure in environments where other domestic ungulates are less productive. Early zootechnical descriptions already emphasized the economic relevance of the yak and of yak × cattle hybrids (“dzo”/“dzomo”) for local husbandry systems [1].

In the past two decades, yaks have attracted renewed interest outside their traditional range, both for niche production systems and as a biological model of adaptation to hypoxia and cold. Genomic resources now confirm that yak and cattle are closely related within the tribe *Bovini*, while showing clear signatures of selection in pathways involved in oxygen transport and energy metabolism [2,3].

Because of their phylogenetic proximity and complementary productive traits, interspecific crosses between yak and cattle have long been attempted to exploit heterosis in growth, milk yield and hardiness. Nonetheless, a consistent reproductive barrier is observed: F1 females are usually fertile, whereas F1 males are typically sterile, which prevents the fixation of favorable combinations through conventional breeding [1,4,5]. The phenomenon has been documented across different production contexts and has been discussed as a paradigmatic case of hybrid male sterility in bovids [6].

Cytogenetic analyses remain essential in livestock not only to define the normal karyotype of a species, but also to support breeding programs by detecting chromosomal abnormalities associated with reduced fertility or embryonic losses. In domestic bovids, classical banding techniques and molecular cytogenetics (notably fluorescence in situ hybridization, FISH) have become routine tools for both clinical diagnostics and research, enabling the identification of subtle structural rearrangements and the integration of physical and genetic maps [7].

At the same time, bovids provide a particularly informative framework for comparative cytogenetics. Despite considerable variation in diploid number among Bovid subfamilies, many *Bovini* species share a conserved autosomal morphology and extensive banding homology. Within domestic bovids, comparisons at the 400–450 band level have shown that autosomes are largely conserved and that the main rearrangements involve Robertsonian fusions and inversions, whereas sex chromosomes may exhibit lineage-specific changes in heterochromatin distribution and gene order [8,9,10].

Against this background, although genomic resources for yak have expanded substantially through chromosome-scale assemblies and long-read analysis of structural variation [2,3], the corresponding classical and molecular cytogenetic framework remains comparatively fragmented. The pioneering ideograms produced by Popescu [4] and subsequent comparative studies on heterochromatin and nucleolar organizer regions (NORs) suggested a high level of karyotype conservation with cattle [11]; however, standardized high-resolution banding comparisons and independently validated physical markers linking chromosome morphology to current genome assemblies remain scarce. This gap is relevant because hybrid sterility between yak and cattle arises even in the absence of obvious differences in chromosome number or gross morphology, implying that microstructural divergence or differences in recombination landscapes may be involved.

Beyond gross chromosome morphology, repetitive DNA compartments provide additional information for comparative cytogenetics. In *Bovidae*, constitutive heterochromatin blocks and ribosomal DNA clusters (nucleolar organizer regions, NORs) are among the chromosomal domains that display measurable inter- and intraspecific variation, while still following species-specific patterns [11,12]. Heterochromatin polymorphisms are particularly evident on the Y chromosome and near telomeres and may influence banding appearance without necessarily affecting fitness. For this reason, the combined use of C-banding and NOR detection is valuable for karyotype standardization and for comparing independently generated ideograms.

Another aspect that is often overlooked in non-model livestock species is chromosome stability. In cattle, the frequency of spontaneous chromosome breaks and the rate of sister chromatid exchanges (SCEs) in peripheral blood lymphocytes have been used as indicators of genomic integrity, reflecting both endogenous processes and exposure to environmental stressors or clastogens [13,14]. Chromosome aberration and SCE analyses were therefore included as complementary descriptive analyses of chromosome instability in the examined yak cohort. These measurements were not intended to test environmental acclimatization or to establish species-wide reference.

A leading hypothesis for male sterility in *Bovini* hybrids is that meiotic pairing and recombination between X and Y chromosomes are disrupted. In mammals, correct synapsis of the X and Y chromosomes during male meiosis relies on the pseudoautosomal region (PAR), the only segment where obligatory crossover occurs. The PAR boundary is known to evolve rapidly in cattle and other domestic species [15,16], and comparative cytogenetic mapping indicates that PAR-associated markers are located near the telomeres and close to Y-linked genes such as *ZFY* in several bovids [8,17]. Even small shifts in the PAR boundary or in adjacent heterochromatin can potentially compromise synapsis, leading to meiotic arrest.

Finally, the current genomic era is rapidly expanding resources for yak, including high-quality assemblies and long-read-based surveys of structural variation [2,3]. Nevertheless, sequence-based approaches alone may not resolve large repetitive regions or accurately detect balanced rearrangements, and they benefit from cytogenetic anchoring through chromosome painting and BAC-FISH mapping [7,18]. Integrating classical banding with molecular cytogenetics therefore remains a practical route to connect chromosome-level structure with genome assemblies and, ultimately, with reproductive performance.

Here, we report a cytogenetic investigation of eight domestic yak bulls reared in Italy, combining classical banding approaches (GTG, RBG, RBA and CBA), sequential NOR detection, assessment of chromosome stability (chromosome aberrations and sister chromatid exchanges, SCE) and molecular cytogenetics (zoo-FISH with bovine chromosome paints and FISH mapping of Y-linked genes). In addition, a comparative bioinformatic analysis of available yak genome assemblies was performed to verify their correspondence with the cytogenetic evidence and to identify possible assembly inconsistencies or cryptic genomic differences. We aimed to provide an updated cytogenetic framework for yak, establish cohort-specific exploratory values for chromosome stability in this population, and generate reference information useful for future work on yak improvement programs and on the cytogenetic basis of yak × cattle hybrid sterility.

## 2. Materials and Methods

### 2.1. Ethical Statement

The study protocol followed the provisions of the European Directive 2010/63/EU on the care and use of animals for scientific purposes and was approved by the Ethical Committee of the University of Turin (Protocol No. 0059643—30 January 2023).

### 2.2. Animals and Sampling

Following ethical approval, peripheral blood samples were collected from eight clinically healthy adult male yaks (*B. grunniens*), aged 12–13 years at the time of sampling. The animals belonged to a single ex situ herd established in Central Italy in 2012. They represented all the adult males available. The cohort was not selected to represent the overall genetic diversity of the species. Blood was collected from the tail by sterile heparinized tubes and processed within 24 h.

### 2.3. Lymphocyte Cultures

For each animal, triplicate whole-blood cultures were prepared according to Iannuzzi and Di Berardino [7]. Briefly, RPMI 1640 (Dutch modification) supplemented with 10% fetal bovine serum, 200 mM of L-glutamine (200 mM, Sigma-Aldrich; St. Louis, MO, USA), 1 mg/mL of concanavalin A as mitogen agent and 1× antibiotic-antimycotic solution was used as culture media. Cultures were incubated at 37.5 °C for 72 h. Colcemid (10 μg/μL, Gibco; Grand Island, NY, USA) was added during the last 45 min of culture followed by hypotonic and fixation treatments performed in the usual manner [7]. Cell suspensions were dropped onto clean slides and air-dried. Slides intended for banding and FISH were aged before use according to routine laboratory practice [7].

### 2.4. Chromosome Instabilities

Conventional Giemsa-stained preparations were used to evaluate spontaneous chromosome instability. For each animal, 50 well-spread metaphases (total *n* = 400) were analyzed and chromatid/isochromatid breaks, gaps and acentric fragments were recorded according to standard cytogenetic criteria [7].

### 2.5. Sister Chromatid Exchanges (SCE)

For SCE visualization, 5-bromodeoxyuridine (BrdU; 10 µg/mL final concentration, Sigma-Aldrich; St. Louis, MO, USA) was added 30 h before harvest to allow incorporation during two replication cycles [19]. Sister chromatids were differentially stained with acridine orange. For each animal, 25 second-division metaphases (total *n* = 200) were scored and the mean number of SCEs per cell (±SD) and the observed range were calculated.

### 2.6. Synchronization for High-Resolution Banding

For banded karyotypes, 48 h lymphocyte cultures were synchronized using either thymidine (300 µg/mL final concentration) or methotrexate (0.05 µg/mL final concentration, Sigma-Aldrich, Darmstadt, Germany) for 16 h. The block was released by two washes in fresh medium or Hank’s balanced salt solution. Cultures were then restarted for 5–6 h in the presence or absence of BrdU (15 µg/mL, Sigma-Aldrich; St. Louis, MO, USA) plus Hoechst 33258 (30 µg/mL) to obtain replication banding. Colcemid treatment lasted 30–45 min. Hypotonic and fixation treatments were performed according to the standard cytogenetic method [7].

### 2.7. Banding and NOR Techniques

GTG banding was obtained by brief trypsin treatment followed by Giemsa staining [20]. Replication banding (RBG and RBA) and C-banding were performed without modification according to the established protocols described in the literature [7,21]. Nucleolar organizer regions (NORs) were detected by silver staining (Ag-NOR) [22] on RBA-banded preparations to assign NORs to specific chromosomes. Banded karyotypes and ideograms were arranged according to the International System for Chromosome Nomenclature of Domestic Bovids (ISCNDB) [23].

### 2.8. Zoo-FISH and BAC-FISH Mapping

Whole chromosome painting probes obtained from microdissected bovine chromosomes X, Y, 5 and 15 were used for comparative zoo-FISH on yak prometaphases. The autosomal painting probes were selected according to two complementary criteria. The BTA5 probe, which was known to yield a strong chromosome-specific signal [24,25], was included as a technical reference for the efficiency and specificity of the zoo-FISH. The BTA15 probe was selected because, in a subset of banded prometaphases, the correspondence of the putative yak chromosome 15 with the standard cattle chromosome 15 was not always unequivocal. The BTA15 paint was therefore used to confirm chromosome assignment and to investigate whether this chromosome showed any translocation to other yak chromosomes. In addition, bovine BAC clones containing the Y-linked genes *ZFY* and *SRY* were used for FISH mapping on the yak Y chromosome. Probes were labeled by DOP-PCR and nick translation for painting and gene mapping, respectively, using biotin- or digoxigenin-conjugated nucleotides and hybridized to denatured chromosome spreads in the presence of competitor DNA, applied without modification as described in the literature [26]. After overnight hybridization at 37 °C, stringent post-hybridization washes were performed and probes were detected with avidin/anti-avidin or anti-digoxigenin fluorochrome conjugates. Slides were counterstained with DAPI or propidium iodide (Sigma-Aldrich; St. Louis, MO, USA) and mounted in antifade medium (Vector Laboratories, Newark, CA, USA) [18,26,27].

### 2.9. Microscopy and Image Analysis

Metaphases were examined under a Zeiss Axio Imager M2 epifluorescence microscope (Zeiss, Oberkochen, Germany) and equipped with a high-resolution CoolCube1M camera (Zeiss, Oberkochen, Germany). ISIS and Ikaros karyo M ver 7 software from Metasystem were used, respectively, for FISH image acquisition and processing and for karyotyping.

### 2.10. Comparative Genomic Analysis

Available *B. grunniens* genome assemblies were retrieved from the NCBI Assembly database and compared based on accession number, genome size, chromosome representation, sequencing coverage and sequencing technology. For comparative genomic analysis, the chromosome-level *B. grunniens* Bosgru_T2T1.0 assembly was compared with the *B. taurus* ARS-UCD2.0 reference genome using the NCBI Comparative Genome Viewer (CGV). Whole-genome alignments were inspected for the 29 autosomes and the X chromosome, with particular attention paid to chromosome orientation, large-scale correspondence between homologous chromosomes and local inconsistencies detectable at a minimum resolution of 100,000 bp. The BosGru3.1 assembly was also inspected for comparison because it is currently viewable through the NCBI Genome Viewer.

## 3. Results and Discussion

### 3.1. GTG-RBG-RBA-CBA-Banding Patterns

**Figure 1:**
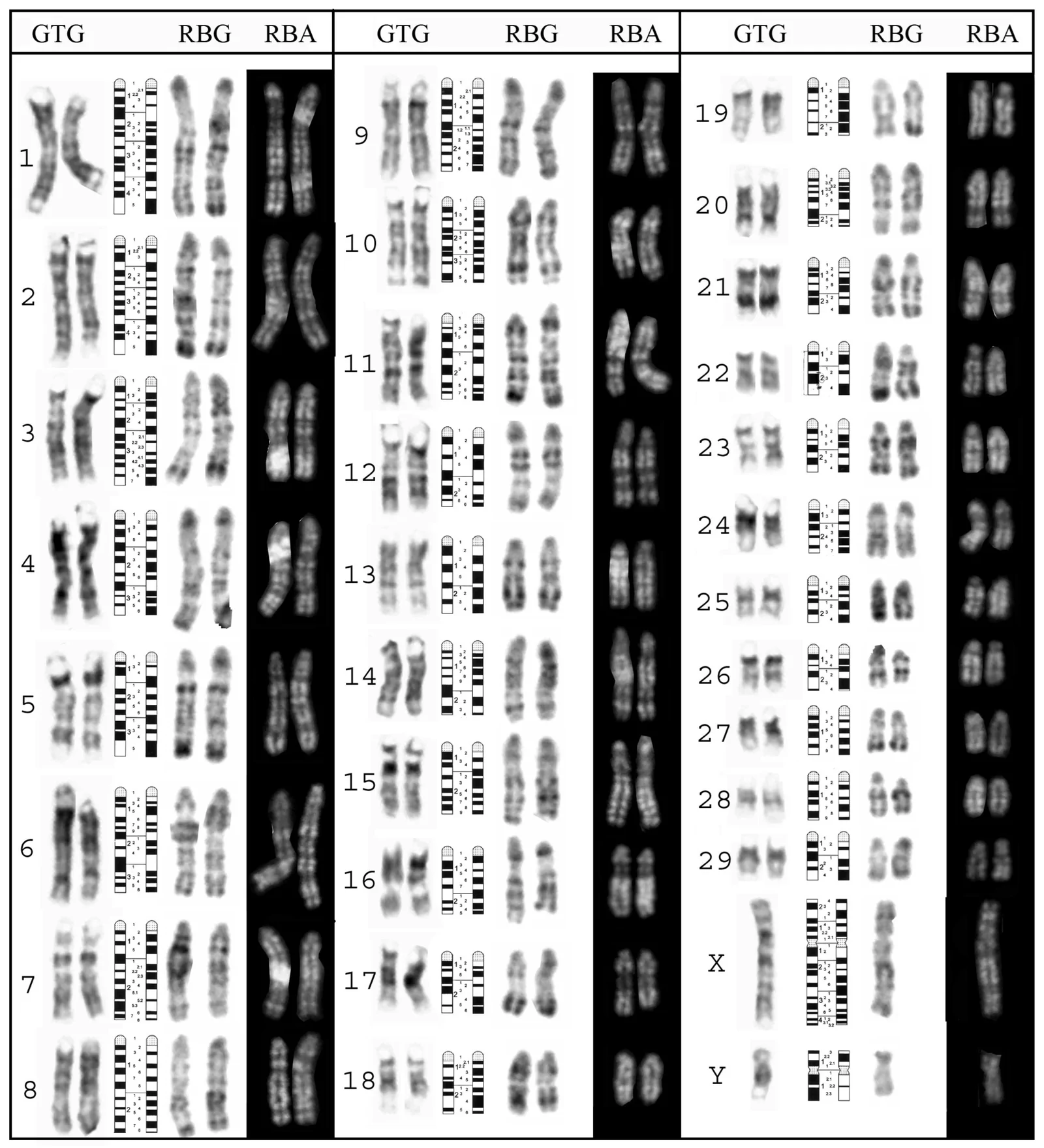
GTG-, RBG- and RBA-banding pattern, at 400-band level, of yak chromosomes and their comparison with standard ideogram of cattle chromosomes [23].

Figure 1 shows, side by side, the GTG-RBG and RBA-banded chromosomes of yak at the 400-band level, all arranged according to ISCNDB 2000 [23].

In between the GTG- and RBG-banding, the standardized ideogram of cattle is reported for direct comparison. At this level of resolution, all yak chromosomes were found to be largely similar to those of cattle.

**Figure 2:**
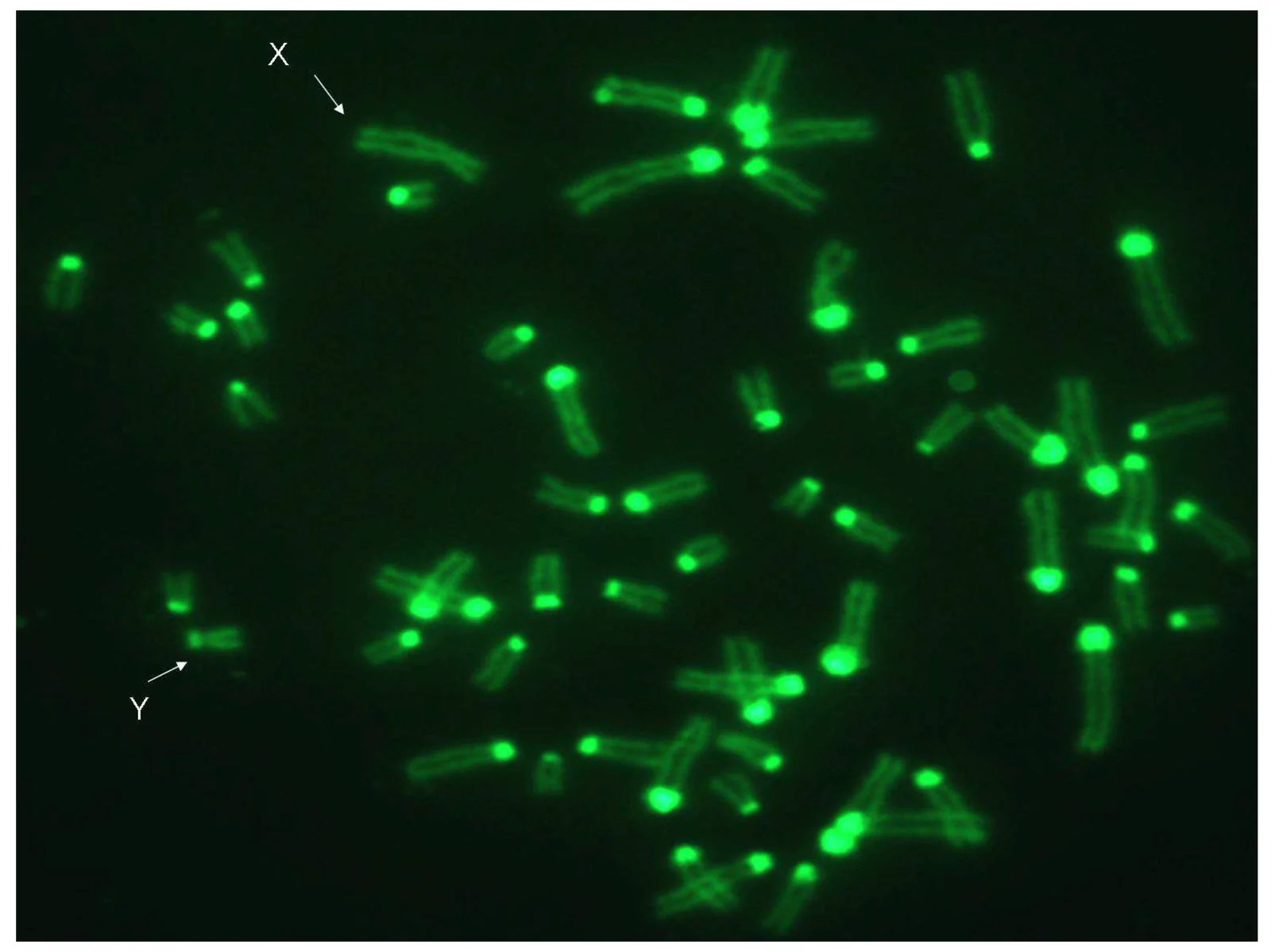
CBA-banded metaphase spread of the yak with indication of sex chromosomes.

Figure 2 shows the CBA-banded metaphase. The chromosomes were arranged according to decreasing size. As in other bovids, the X chromosome was entirely C-negative, whereas the Y chromosome showed prominent C-positive blocks in its distal region. The amount of constitutive heterochromatin appeared variable among individuals and between homologous autosomal pairs, consistent with the heterochromatin polymorphism described in *Bovidae* [8,11,12].

### 3.2. Chromosome Instabilities

**Figure 3:**
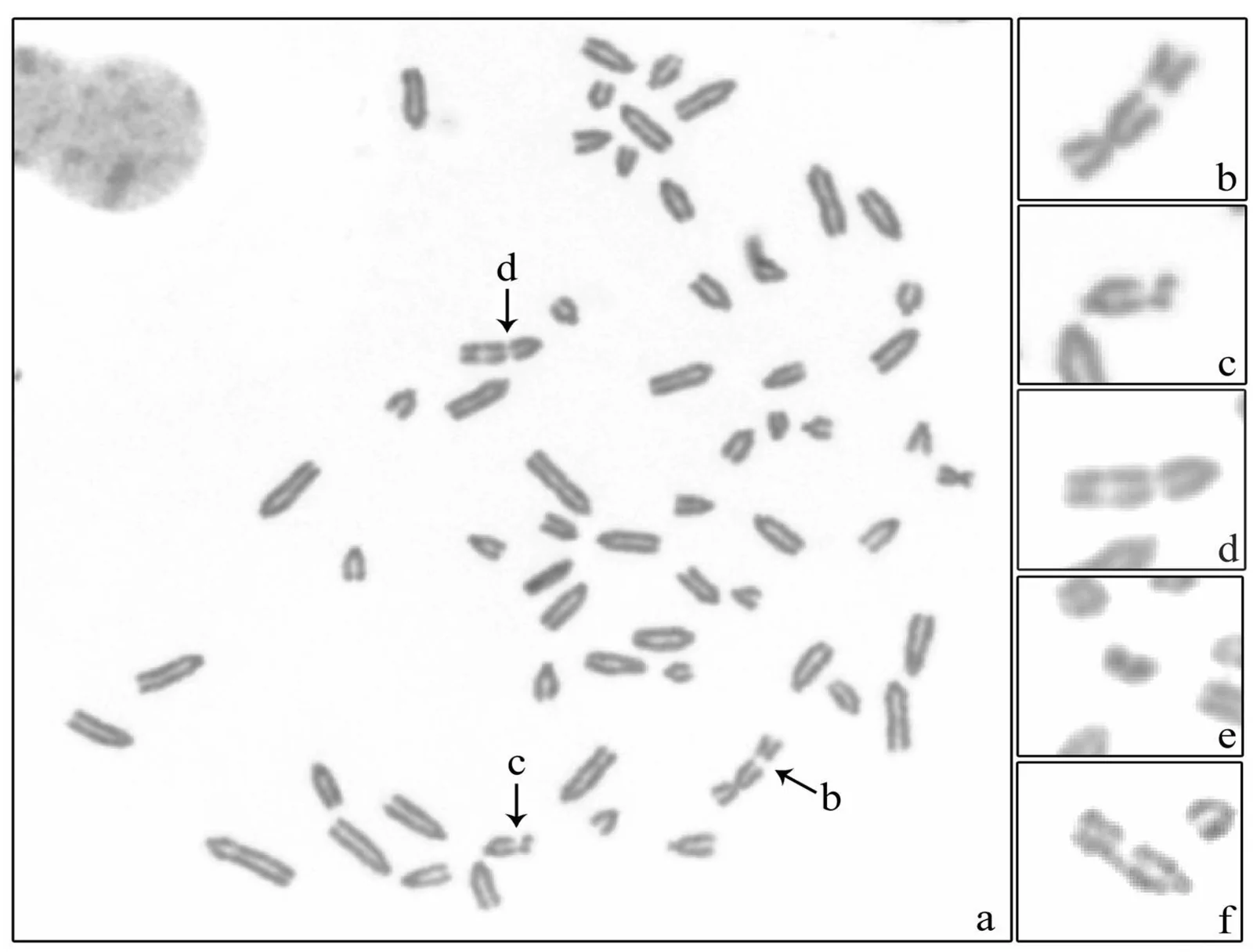
(**a**) Chromosome aberrations in conventionally Giemsa-stained metaphase plate of yak with chromosomal abnormalities; (**b**) iso-chromatid break on the X chromosome; (**c**) chromatid break; (**d**) iso-chromatid break and gap; (**e**) chromosome fragment; and (**f**) chromatid break.

Figure 3a and Table 1 show the various types of chromosome aberrations found in the sample examined. Out of 400 cells examined (50 for each animal), 15 were found to be abnormal (3.75%).

Of these, iso-chromatid plus chromatid breaks were the most frequent (77.8%), followed by gaps (17.4%) and fragments (4.4%). In a similar study on 200 cells in cattle, Di Berardino et al. [13] reported an incidence of 3.0% of abnormal cells in a control group of four healthy cows based on the analysis of 50 metaphases per animal, with chromatid and iso-chromatid breaks accounting for 67% of the recorded abnormalities.

### 3.3. Sister Chromatid Exchanges (SCEs)

**Figure 4:**
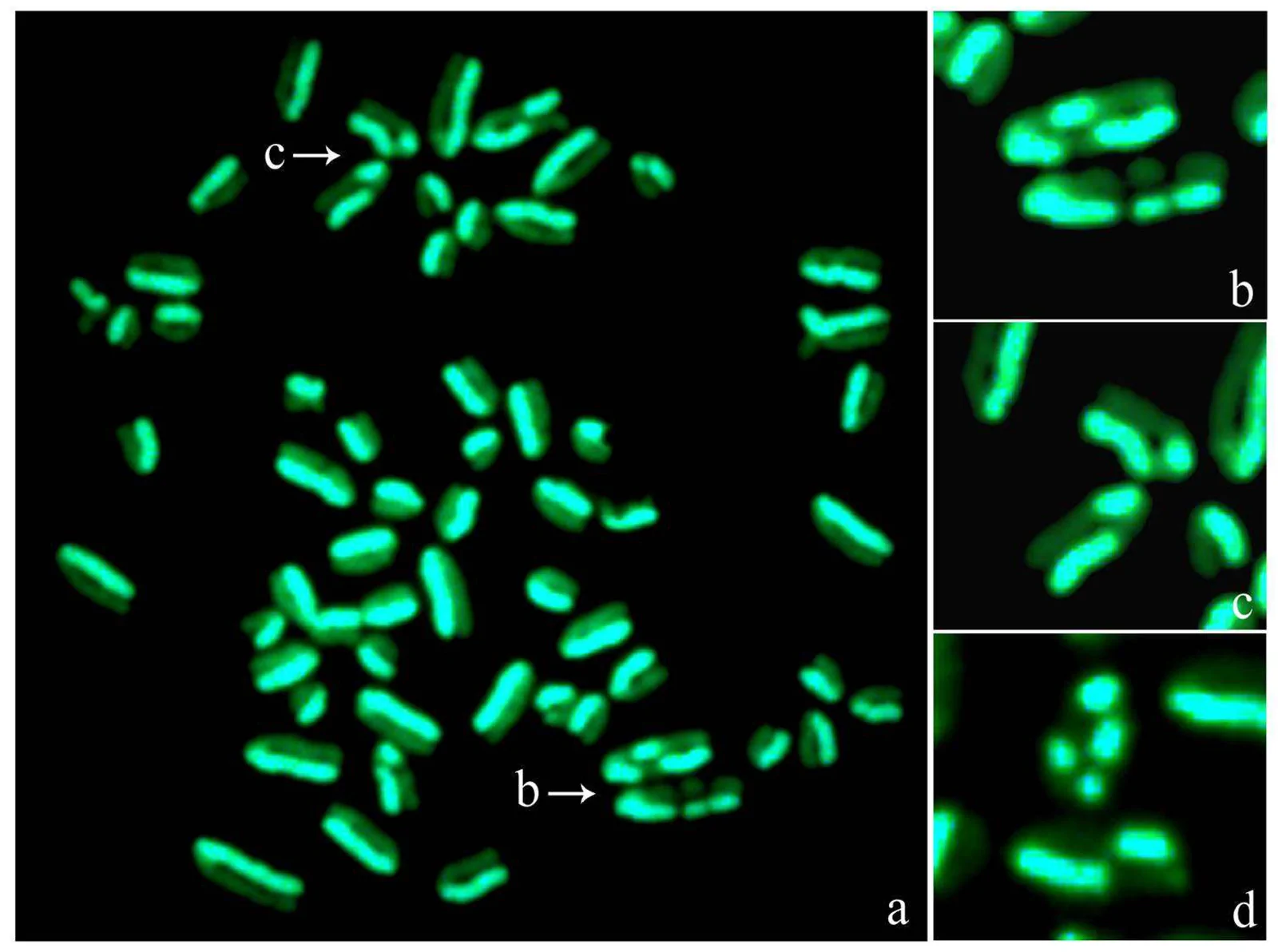
Sister chromatid exchanges (SCEs) in yak chromosomes: (**a**) second-division metaphase plate; (**b**–**d**) examples of SCE events on autosomes and on the X chromosome (arrows).

Figure 4 and Table 2 show the sister chromatid exchanges in the yak chromosomes. A total of 200 sister chromatid differentially stained cells were examined (25 for each animal).

The mean rate of SCE per cell was 5.19 ± 2.14, with a range from 1 to 13. In cattle lymphocytes cultured under comparable BrdU conditions, a mean SCE frequency in the range of 5.4 ± 2.1 per cell has been reported in former and more recent studies [13,19].

### 3.4. Nucleolus Organizer Regions (NORs)

**Figure 5:**
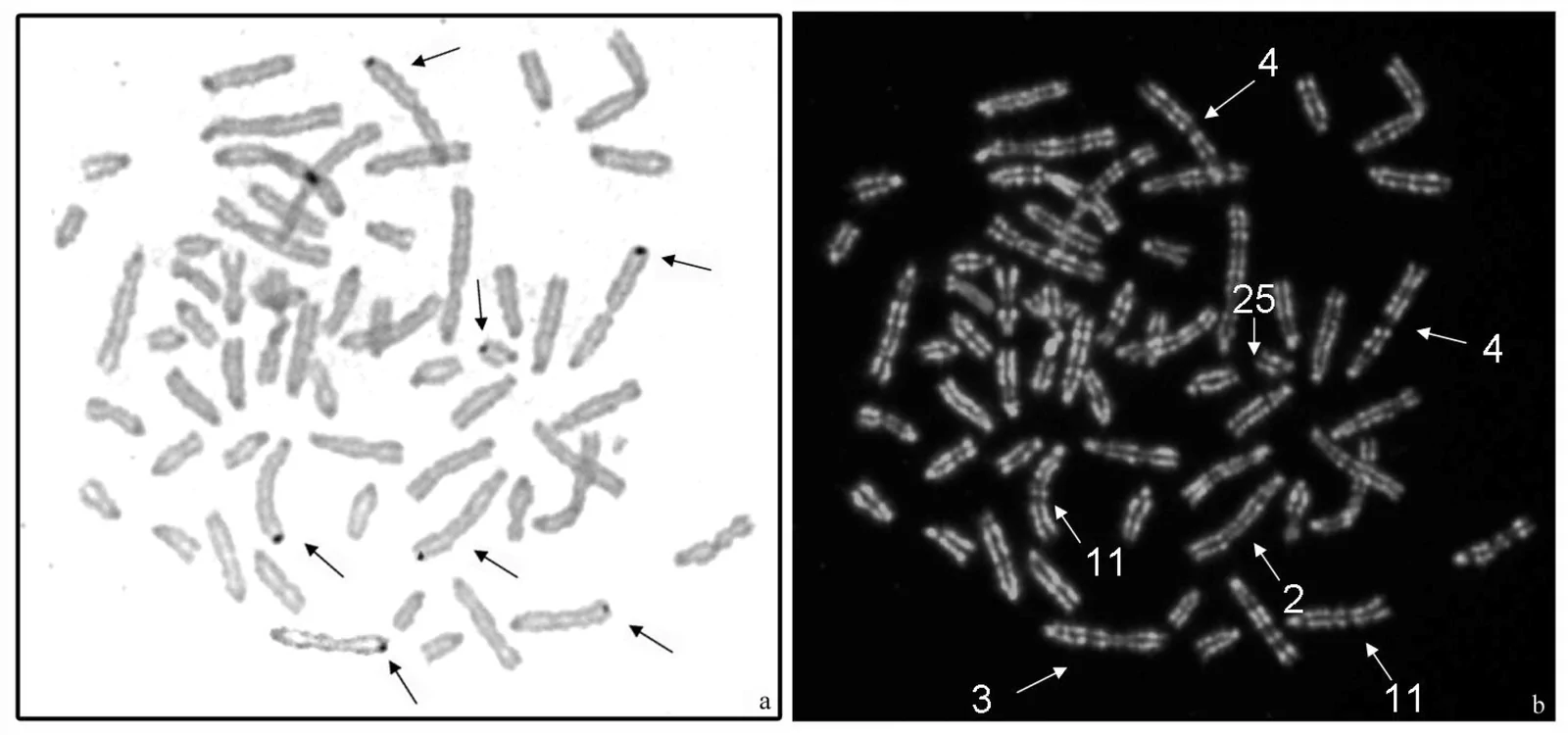
Sequential RBA + NOR staining: (**a**) AgNO_3_ staining identified the telomeric NORs (arrows); (**b**) RBA localized the NORs on autosomes 2, 3, 4, 11 and 25 (arrows).

Figure 5 shows the sequential NOR + RBA-banding technique. The NORs are shown in (a) while the RBA-banding is shown in (b). NORs were localized on the telomeres of chromosomes 2, 3, 4, 11 and 25 of the ISCNDB 2000 standardized karyotype, matching the pattern described for domestic cattle [11,12].

### 3.5. Zoo-FISH and Gene Mapping

**Figure 6:**
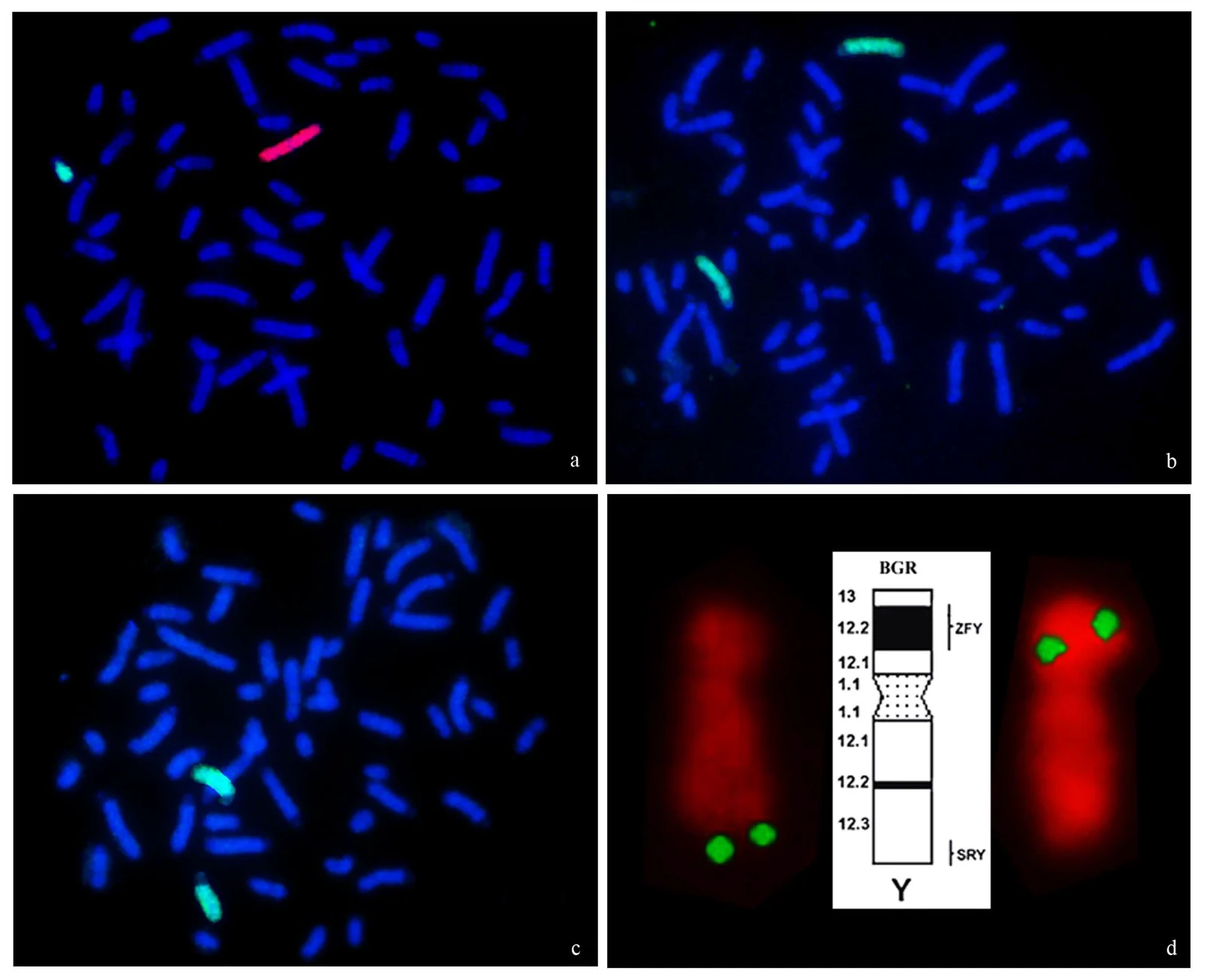
Zoo-FISH on yak chromosomes, performed by using painting probes from bovine chromosomes (**a**) X (red color) and Y (green color), (**b**) 5 (green), and (**c**) 15 (green). (**d**) FISH mapping of *ZFY* and *SRY* genes (both green) on the yak Y chromosome (red by propidium iodide staining).

Figure 6 shows the fluorescent in situ hybridization (zoo-FISH) on yak metaphase chromosomes by using painting probes from cattle chromosomes X-Y (a), 5 (b), and 15 (c).

The bovine painting probes hybridized entirely to the homologous chromosomes of the yak, thus indicating close homology between the two species. In particular, the BTA5 painting probe produced the expected strong and chromosome-specific signal, confirming the technical efficiency of the cross-species hybridization as for the sex chromosomes paintings. The BTA15 probe hybridized completely and exclusively to the homologous yak chromosome 15, with no detectable additional signals on other chromosomes. This result provided no evidence of an interchromosomal rearrangement involving BTA15 homologous sequences. On the other hand, the apparent banding variability observed in some preparations cannot be fully resolved by whole-chromosome painting, which does not allow the detection of small intrachromosomal rearrangements, including inversions, or fine-scale sequence differences.

The FISH mapping of bovine BAC clones containing *ZFY* and *SRY* genes on the yak Y chromosome is shown in Figure 6d. The two genes were localized in the same positions reported in cattle (Yp12.2 and Yq23dist, respectively) [8].

Overall, the yak karyotype observed here (2n = 60. NF = 62) is highly conserved relative to cattle, confirming the extensive chromosomal conservation within *Bovini*. GTG/RBG/RBA patterns did not show reproducible differences at the 400-band level from the bovine standard, and chromosome paints from BTA X, Y, 5 and 15 hybridized entirely to their yak homologues. This concordance is consistent with earlier comparative ideograms [4] and with broad banding homologies documented among domestic bovids [9].

CBA banding revealed the expected distribution of constitutive heterochromatin, with the X chromosome largely being C-negative and the Y chromosome characterized by a distal C-positive block. Variability in the size of heterochromatin blocks is common in bovids and can modify the apparent morphology of sex chromosomes without necessarily implying functional differences [8,11]. This has also been confirmed more recently in the Mediterranean river buffalo by next-generation sequencing data on the Y chromosome where variability has been discovered in 10 bulls both in terms of gene copy and the size of heterochromatin blocks [28].

Sequential Ag-NOR/RBA staining confirmed five NOR-bearing autosomal pairs (2, 3, 4, 11 and 25), matching the bovine pattern [11,12] and providing useful landmarks for karyotype standardization.

At the resolution of the approaches used, no reproducible large-scale chromosomal rearrangements were detected. These results do not exclude smaller balanced rearrangements, copy number variation (CNV), sequence divergence or differences involving highly repetitive and pseudoautosomal regions.

With regard to chromosome stability, the incidence of spontaneous aberrant metaphases in our sample (3.75%) fell within the range reported for clinically healthy cattle lymphocytes evaluated with similar criteria [13]; the observed SCE rate (5.19 ± 2.14) was also within the range reported in cattle [14,19]. These findings should be interpreted separately from the constitutional karyotype data consistently observed across the examined animals; spontaneous chromosome aberration and SCE may be influenced by age, health status, culture conditions and cumulative exposure history. Therefore, in the absence of a concurrently examined native yak group, our data describe this specific yak stock, and no effect of rearing under Italian conditions can be inferred. Furthermore, our data cannot exclude the fact that very rare events, low-level mosaicism, or abnormalities may occur in a wider population. Despite this, these cohort-specific chromosomal instability values may support future cytogenetic monitoring of yak breeding stocks.

The FISH localization of bovine BAC clones containing *ZFY* and *SRY* to Yp12.2 and Yq23dist on the yak Y chromosome adds two molecular landmarks to the yak karyotype and further underscores the conservation of the Y chromosome gene order across bovids [8]. Nevertheless, the remarkable chromosomal similarity observed here also highlights a key biological paradox: despite a conserved chromosome number and large-scale homology, male hybrids between yak and cattle are typically sterile [1,4,5,6]. Whole-chromosome painting is not expected to detect small inversions, CNV in repetitive regions, or shifts in recombination boundaries that could affect meiosis and fertility.

One plausible target is the pseudoautosomal region (PAR), which must undergo pairing and at least one crossover between X and Y during male meiosis. In cattle, the PAR boundary has been characterized at the sequence level and shown to have a complex evolutionary history [15,16,29]. Comparative mapping across bovids indicates that PAR-associated markers are located near telomeric regions and close to Y-linked genes such as *ZFY* [8,17]. Even minor divergence in PAR size, boundary position or surrounding heterochromatin could compromise X–Y synapsis and trigger meiotic arrest, providing a mechanistic route to hybrid male sterility. Future work combining high-resolution BAC-FISH across the PAR boundary, long-read assemblies of the yak sex chromosomes, and direct analyses of meiotic pairing in yak × cattle hybrids would be particularly informative.

In other interspecific crosses within *Bovidae*, reproductive competence is strongly sex-dependent, with male offspring generally being sterile, whereas females often retain fertility [30]. Instead, crosses between taurine and indicine cattle represent a notable exception, because *B. taurus × B. indicus* offspring of both sexes are normally fertile [31]. This finding is particularly relevant given that the two taxa differ in the morphology and organization of the Y chromosome [32]. Moreover, analyses of synaptonemal complexes in male *B. taurus × B. indicus* hybrids have revealed an increased frequency of meiotic chromosome-pairing irregularities [33]. In particular, the latter study demonstrated that size heteromorphism of the centromeric heterochromatin blocks resulted in occasional nonspecific associations between the sex bivalent (X–Y) and an autosomal bivalent containing an unsynapsed pericentromeric region. Although these associations were observed only in a small proportion of primary spermatocytes, they may have contributed to the reduced fertility of hybrid bulls. As similar meiotic abnormalities could occur in yak × cattle hybrids, the synaptonemal complex (SC) analysis that enables the evaluation of chromosome pairing during the pachytene substage of meiotic prophase I could be particularly useful for understanding, at least partially, the reason of hybrid males’ infertility.

### 3.6. Comparative Genomic Analysis of Yak and Cattle Assemblies

The cytogenetic data of this study show a broad chromosome-scale correspondence between the chromosomes of *B. taurus* and *B.* *grunniens*, a correspondence that could be explained by the recent phylogenetic divergence between the two species, dating back to approximately 3.3–5.0 million years ago [34] during the Pliocene. However, within the Bovidae family, these similarities suggested by cytogenetics have sometimes been partially refuted by more detailed genomic analyses, which go beyond the definition provided by cytogenetic analyses. Comparative cytogenetic analysis between cattle (*B. taurus*) and water buffalo (*Bubalus bubalis*), species that diverged phylogenetically approximately 8.2–10.9 million years ago in the late Miocene, suggests the existence of only five centromeric fusions [35], whereas genomic analyses have recently revealed the existence of two inversions not detectable by standard cytogenetic analyses [36]. Other cryptic differences have been identified between cattle (*B. taurus*) and goats (*Capra hircus*) [37], between cattle (*B. taurus*) and sheep (*Ovis aries*) [38], and between the X chromosomes of cattle (*B. taurus*) and goats (*C. hircus*) [39].

Therefore, to complement the cytogenetic comparison, we inspected the currently available genome assemblies of *B. grunniens* and conducted a genomic-level analysis of the similarities with cattle (*B. taurus*). Five yak assemblies are available, differing in genome size, sequencing technology, coverage and chromosome representation (Table 3).

Most assemblies include the autosomes and the X chromosome, whereas BosGru3.1 also includes the Y chromosome and SMU_DYY_1.0 is restricted to the Y chromosome. Among them, Bosgru_T2T1.0 is assigned reference status by NCBI, while BosGru3.1 is currently viewable through the NCBI Genome Viewer [2,40].

**Figure 7:**
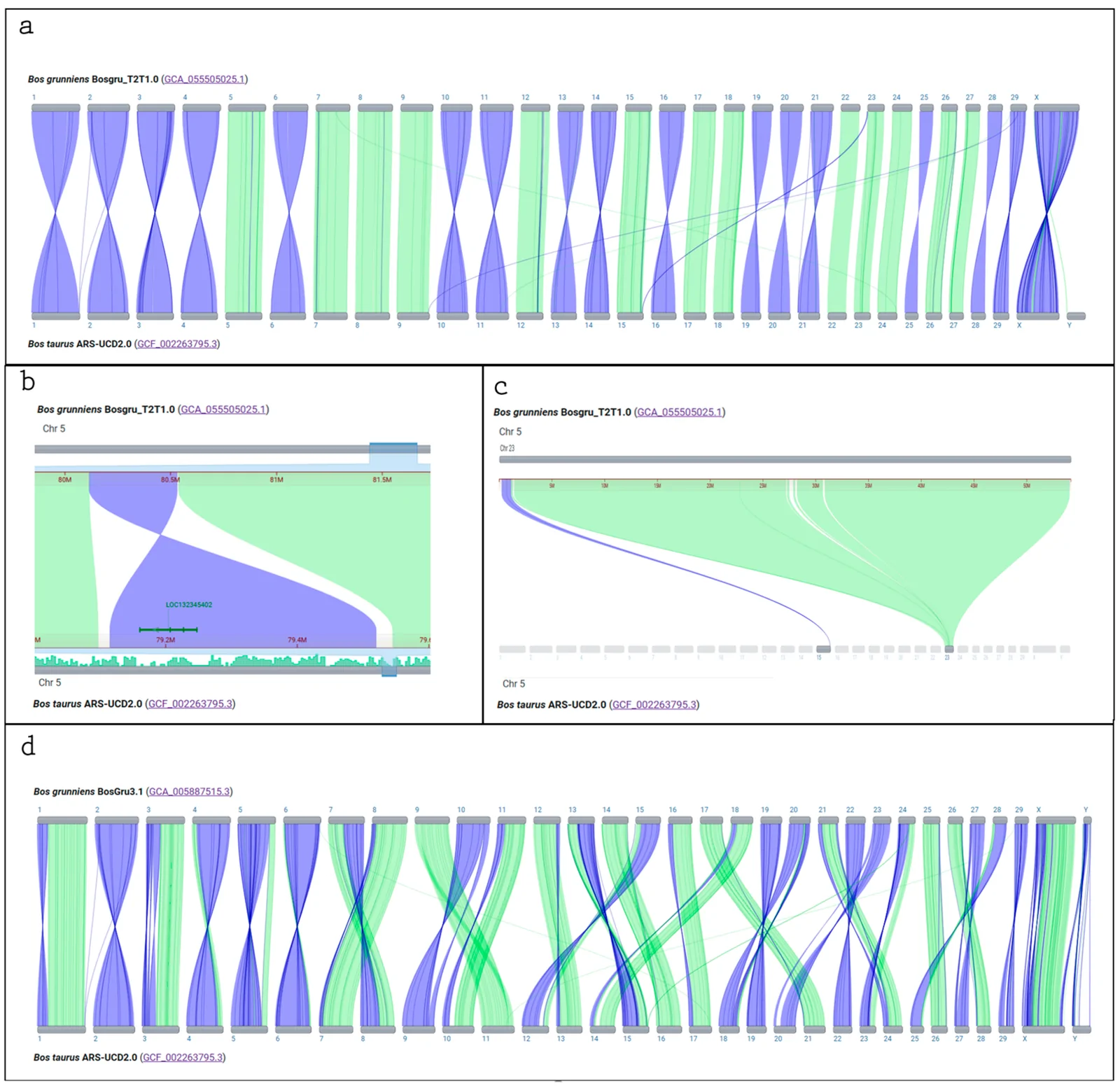
(**a**) Visualization using CGV of the similarities between the 29 autosomes and the X chromosome in *B. grunniens* (Bosgru_T2T1.0 assembly) and *B. taurus* (ARS_UCD2.0). (**b**) Detail of the inversion between BGRU5 and BTA5. (**c**) Detail of the transposition between BGRU23 and BTA15. (**d**) Visualization using CGV of the similarities between the 29 autosomes and the X chromosome in *B. grunniens* (BosGru3.1) and *B. taurus* (ARS_UCD2.0). All analyses were conducted with a minimum resolution of 100,000 bp. Green ribbons indicate aligned regions in the same relative orientation, whereas blue ribbons indicate alignments in reverse orientation between the two assemblies.

The comparative analysis of the two genomes (*B. taurus* ARS-UCD2.0 and *B. grunniens* Bosgru_T2T1.0), performed using the Comparative Genome Viewer tool [41], shows excellent alignment (Figure 7a). However, it should be noted that more than several yak chromosome sequences were displayed in the opposite coordinate orientation relative to their cattle homologues. This does not, by itself, indicate an evolutionary inversion, but it should be considered when genomic coordinates are used to select locus-specific probes, for example, to perform FISH analysis.

Furthermore, when analyzing similarities at a resolution of 100,000 bp, it is possible to detect small inconsistencies, such as an inversion of approximately 0.5 Mb affecting chromosomes BGRU5/BTA5 (Figure 7b), or the presence of a centromeric region of approximately 1 Mb on BGRU23 that maps to BTA15 (Figure 7c). These local discordances remain candidate differences or assembly inconsistencies that require orthogonal validation by BAC-FISH or read-level genomic analyses [39].

It should be noted that this assembly shows a significant improvement over the one currently available in the NCBI Genome Viewer (BosGru3.1, Figure 7d), which contained numerous inconsistencies with the results of the karyotype analysis.

## 4. Conclusions

This study provides an updated cytogenetic characterization of the domestic yak reared in Italy, integrating classical chromosome banding, chromosome instability assessment, NOR detection, chromosome painting and BAC-FISH mapping, and comparative genome analysis. At the approximately 400-band level, no reproducible large-scale differences were detected, and the results confirm the highly conserved organization of the yak karyotype in comparison with cattle, providing useful reference information for cytogenetics standardization in this species. This result is restricted to the resolution of the approaches used and does not exclude smaller balanced rearrangements, copy number variation or divergence within repetitive and PARs. At the same time, bioinformatics analysis of genomes highlighted chromosome orientation issues and local discrepancies that should be considered when using yak assemblies for comparative mapping or probe design. Furthermore, our results highlight the need for higher-resolution investigations to explain the reproductive barriers observed in yak × cattle hybrids. Future studies should therefore focus on fine-scale structural variation, using integrated cytogenetic and genomic approaches.

## Figures and Tables

**Table 1:** Numerical and percentage distribution of chromosomal abnormalities in the cell population of the eight yak bulls examined.

N	Cells			Type of Abnormality								
	Examined N (1)	Abnormal		Total	Breaks				Gaps		Fragment	
					Chromatid.		Iso-Cromat.					
		N	% on (1)	N (2)	N	% on (2)	N	% on (2)	N	% on (2)	N	% on (2)
1	50	2	4.0	2	1	50.0	1	50.0	-	-	-	-
2	50	1	2.0	1	1	100.0	-	-	-	-	-	-
3	50	2	4.0	3	-	-	2	66.7	1	33.3	-	-
4	50	-	-	-	-	-	-	-	-	-	-	-
5	50	2	4.0	4	1	25.0	2	50.0	-	-	1	25.0
6	50	4	8.0	6	2	33.3	3	50.0	1	16.7	-	-
7	50	2	4.0	4	1	25.0	1	25.0	2	50.0	-	-
8	50	2	4.0	3	1	33.3	2	66.7	-	-	-	-
	400	15	3.75	23	7	30.4	11	47.8	4	17.4	1	4.4

**Table 2:** Mean sister chromatid exchange (SCE) rate per cell in the examined yak samples.

Animals	Cells Examined	SCE/cell	
N.	N.	Mean ± SD	Range
1	25	5.28 ± 2.38	2–10
2	25	5.00 ± 1.79	1–8
3	25	5.72 ± 2.54	1–13
4	25	5.00 ± 1.69	1–8
5	25	5.04 ± 1.87	4–9
6	25	4.96 ± 2.13	3–9
7	25	5.40 ± 2.24	1–9
8	25	5.08 ± 2.25	2–9
Total	200	5.19 ± 2.14	1–13

**Table 3:** Characteristics of the 5 genomic assemblies currently available for the yak.

	Bosgru_T2T1.0	NRCY_Bgru-v1	NWIPB_DYAL_1.0	BosGru3.1	SMU_DYY_1.0
GenBank	GCA_055505025.1	GCA_050059765.1	GCA_027580245.1	GCA_005887515.3	GCA_056775595.1
Genome Size	2.8 Gb	2.8 Gb	2.6 Gb	2.8 Gb	42.4 Mb
Chromosome	Autosomes + X	Autosomes + X	Autosomes + X	Autosomes + XY	Y
Genome Coverage	64×	30.47×	253.06×	239×	100×
Seq. technology	Oxford Nanopore	PacBio Sequel	Oxford Nanopore Illumina HiSeq	Oxford Nanopore Illumina HiSeq	PacBio Sequel

## Data Availability

The original contributions presented in this study are included in the article. Further inquiries can be directed to the corresponding author.

## Abbreviations

The following abbreviations are used in this manuscript:

BAC	Bacterial artificial chromosome
BGRU	*Bos grunniens*
BTA	*Bos taurus*
CBA	C-banding by acridine orange
CNV	Copy number variation
GTG	G-banding by trypsin Giemsa
FISH	Fluorescence in situ hybridization
PAR	Pseudoautosomal region
PCR	Polymerase chain reaction
RBA	Reverse-banding by bromodeoxyuridine and acridine orange
RBG	Reverse-banding by bromodeoxyuridine and Giemsa
NOR	Nucleolar organizer region
SC	Synaptonemal complex
SCE	Sister chromatid exchange

## References

[1] Phillips R.W., Tolstoy I.A., Johnson R.G. (1946). Yaks and yak-cattle hybrids in Asia. J. Hered..

[2] Qiu Q., Zhang G., Ma T., Qian W., Wang J., Ye Z., Cao C., Hu Q., Kim J., Larkin D.M. (2012). The yak genome and adaptation to life at high altitude. Nat. Genet..

[3] Zhang S., Liu W., Liu X., Du X., Zhang K., Zhang Y., Song Y., Zi Y., Qiu Q., Lenstra J.A. (2021). Structural variants selected during yak domestication inferred from long-read whole-genome sequencing. Mol. Biol. Evol..

[4] Popescu C. (1969). Idiograms of yak (*Bos grunniens*), cattle (*Bos taurus*) and their hybrid. Ann. Génét. Sél. Anim..

[5] Tumennasan K., Tuya T., Hotta Y., Takase H., Speed R., Chandley A. (1997). Fertility investigations in the F1 hybrid and backcross progeny of cattle (*Bos taurus*) and yak (*B. grunniens*) in Mongolia. Cytogenet. Genome Res..

[6] Niayale R., Cui Y., Adzitey F. (2021). Male hybrid sterility in the cattle-yak and other bovines: A review. Biol. Reprod..

[7] Iannuzzi L., Di Berardino D. (2008). Tools of the trade: Diagnostics and research in domestic animal cytogenetics. J. Appl. Genet..

[8] Di Meo G.P., Perucatti A., Floriot S., Incarnato D., Rullo R., Jambrenghi A.C., Ferretti L., Vonghia G., Cribiu E., Eggen A. (2005). Chromosome evolution and improved cytogenetic maps of the Y chromosome in cattle, zebu, river buffalo, sheep and goat. Chromosome Res..

[9] Iannuzzi L., Di Meo G.P. (1995). Chromosomal evolution in bovids: A comparison of cattle, sheep and goat G-and R-banded chromosomes and cytogenetic divergences among cattle, goat and river buffalo sex chromosomes. Chromosome Res..

[10] Iannuzzi L., King W., Di Berardino D. (2009). Chromosome evolution in domestic bovids as revealed by chromosome banding and FISH-mapping techniques. Cytogenet. Genome Res..

[11] Mayr B., Schweizer D., Mendelak M., Krutzler J., Schleger W., Kalat M., Auer H. (1985). Levels of conservation and variation of heterochromatin and nucleolus organizers in the Bovidae. Can. J. Genet. Cytol..

[12] Pathak S., Kieffer N. (1979). Sterility in hybrid cattle: I. Distribution of constitutive heterochromatin and nucleolus organizer regions in somatic and meiotic chromosomes. Cytogenet. Genome Res..

[13] Di Berardino D., Iannuzzi L., Fregola A., Matassino D. (1983). Chromosome instability in a calf affected by congenital malformation. Vet. Rec..

[14] Di Berardino D., Shoffner R. (1979). Sister chromatid exchange in chromosomes of cattle (*Bos taurus*). J. Dairy Sci..

[15] Das P., Chowdhary B., Raudsepp T. (2009). Characterization of the bovine pseudoautosomal region and comparison with sheep, goat, and other mammalian pseudoautosomal regions. Cytogenet. Genome Res..

[16] Van Laere A.-S., Coppieters W., Georges M. (2008). Characterization of the bovine pseudoautosomal boundary: Documenting the evolutionary history of mammalian sex chromosomes. Genome Res..

[17] Raudsepp T., Das P., Avila F., Chowdhary B. (2012). The pseudoautosomal region and sex chromosome aneuploidies in domestic species. Sex. Dev..

[18] Pinkel D., Straume T., Gray J.W. (1986). Cytogenetic analysis using quantitative, high-sensitivity, fluorescence hybridization. Proc. Natl. Acad. Sci. USA.

[19] Pauciullo A., Gaspa G., Genualdo V., Rossetti C., Perucatti A., Milanese G., Gini M.A., Caserta F., Rastello L., Gerbelle M. (2026). Genome Integrity in Dairy Cows Fed Black Soldier Fly Oil: An Integrated Sister Chromatid Exchange and Alkaline Comet In Vivo Assessment. Genes.

[20] Seabright M.A. (1971). A rapid banding technique for human chromosomes. Lancet.

[21] Sumner A. (1972). A simple technique for demonstrating centromeric heterochromatin. Exp. Cell Res..

[22] Goodpasture C., Bloom S.E. (1975). Visualization of nucleolar organizer regions in mammalian chromosomes using silver staining. Chromosoma.

[23] Di Berardino D., Di Meo G., Gallagher D., Hayes H., Iannuzzi L. (2001). International system for chromosome nomenclature of domestic bovids (ISCNDB 2000). Cytogenet. Cell Genet..

[24] Nicodemo D., Pauciullo A., Cosenza G., Peretti V., Perucatti A., Di Meo G., Ramunno L., Iannuzzi L., Rubes J., Di Berardino D. (2010). Frequency of aneuploidy in in vitro–matured MII oocytes and corresponding first polar bodies in two dairy cattle (*Bos taurus*) breeds as determined by dual-color fluorescent in situ hybridization. Theriogenology.

[25] Pauciullo A., Nicodemo D., Cosenza G., Peretti V., Iannuzzi A., Di Meo G., Ramunno L., Iannuzzi L., Rubes J., Di Berardino D. (2012). Similar rates of chromosomal aberrant secondary oocytes in two indigenous cattle (*Bos taurus*) breeds as determined by dual-color FISH. Theriogenology.

[26] Pauciullo A., Perucatti A., Cosenza G., Iannuzzi A., Incarnato D., Genualdo V., Di Berardino D., Iannuzzi L. (2014). Sequential cross-species chromosome painting among river buffalo, cattle, sheep and goat: A useful tool for chromosome abnormalities diagnosis within the family Bovidae. PLoS ONE.

[27] Kubickova S., Cernohorska H., Musilova P., Rubes J. (2002). The use of laser microdissection for the preparation of chromosome-specific painting probes in farm animals. Chromosome Res..

[28] Pauciullo A., Letaief N., Ala U., Černohorská H., Kubičková S., Vozdová M., Perucatti A., Iannuzzi L., Gaspa G., Zhang Y. (2026). Isolation and Sequencing of the Y Chromosome in Mediterranean River Buffalo Using Laser Microdissection-Based NGS. Genes.

[29] Liu R., Low W.Y., Tearle R., Koren S., Ghurye J., Rhie A., Phillippy A.M., Rosen B.D., Bickhart D.M., Smith T.P. (2019). New insights into mammalian sex chromosome structure and evolution using high-quality sequences from bovine X and Y chromosomes. BMC Genom..

[30] Switonski M., Stranzinger G. (1998). Studies of synaptonemal complexes in farm mammals-a review. J. Hered..

[31] Gray A.P. (1972). Mammalian Hybrids: A Check-List with Bibliography. Technical Communications. Commonwealth Bureau of Animal Breeding and Genetics.

[32] Goldammer T., Brunner R., Schwerin M. (1997). Comparative analysis of Y chromosome structure in *Bos taurus* and *B. indicus* by FISH using region-specific, microdissected, and locus-specific DNA probes. Cytogenet. Genome Res..

[33] Switonski M., Ansari H., Mathew A., Jung H., Stranzinger G. (1990). Synaptonemal complex analysis in primary spermatocytes of cattle x zebu hybrids (*Bos taurus* x *Bos indicus*). J. Anim. Breed. Genet..

[34] Hassanin A., Delsuc F., Ropiquet A., Hammer C., Van Vuuren B.J., Matthee C., Ruiz-Garcia M., Catzeflis F., Areskoug V., Nguyen T.T. (2012). Pattern and timing of diversification of Cetartiodactyla (Mammalia, Laurasiatheria), as revealed by a comprehensive analysis of mitochondrial genomes. Comptes Rendus Biol..

[35] Iannuzzi L., Di Meo G., Perucatti A., Schibler L., Incarnato D., Gallagher D., Eggen A., Ferretti L., Cribiu E., Womack J. (2003). The river buffalo (*Bubalus bubalis*, 2n= 50) cytogenetic map: Assignment of 64 loci by fluorescence in situ hybridization and R-banding. Cytogenet. Genome Res..

[36] Pistucci R., Cascone I., Iannuzzi A., Albarella S., Kowal-Mierzwa W., Zannotti M., Iannuzzi L., Parma P. (2025). Comparative analysis of cattle (*Bos taurus*, 2n = 60) and river buffalo (*Bubalus bubalis*, 2n = 50) genome assemblies reveals two evolutionary conserved inversions and invalid centromere–telomere orientation of some autosomes. Anim. Genet..

[37] De Lorenzi L., Planas J., Rossi E., Malagutti L., Parma P. (2015). New cryptic karyotypic differences between cattle (*Bos taurus*) and goat (*Capra hircus*). Chromosome Res..

[38] De Lorenzi L., Pauciullo A., Iannuzzi A., Parma P. (2020). Cytogenetic characterization of a small evolutionary rearrangement involving chromosomes BTA21 and OAR18 *Cytogenet*. Genome Res..

[39] Kowal-Mierzwa W., Pistuccci R., Iannuzzi A., Zannotti M., Iannuzzi L., Bugno-Poniewierska M., Parma P. (2026). Identification of Cryptic Evolutionary Rearrangements Between Cattle (*Bovinae*) and Goat (*Caprinae*) X Chromosomes. Anim. Genet..

[40] Liu Y., Luo J., Dou J., Yan B., Ren Q., Tang B., Wang K., Qiu Q. (2020). The sequence and de novo assembly of the wild yak genome. Sci. Data.

[41] Rangwala S.H., Rudnev D.V., Ananiev V.V., Oh D.-H., Asztalos A., Benica B., Borodin E.A., Bouk N., Evgeniev V.I., Kodali V.K. (2024). The NCBI Comparative Genome Viewer (CGV) is an interactive visualization tool for the analysis of whole-genome eukaryotic alignments. PLoS Biol..

